# Preparation, Structural Characterization and Biological Activity Study of Selenium-Rich Polysaccharides from *Cyclocarya paliurus*

**DOI:** 10.3390/foods14091641

**Published:** 2025-05-07

**Authors:** Yulan Dong, Zijue Wang, Qinghui Xia, Juan Chen, Quanwei Lv, Shaopeng Zhang, Shuiyuan Cheng, Xiaoling Chen, Xingxing Dong

**Affiliations:** 1 School of Modern Industry for Selenium Science and Engineering, Wuhan Polytechnic University, Wuhan 430023, China; 17603983279@163.com (Y.D.); 13039522428@163.com (Z.W.); 15571778723@163.com (Q.X.); 13438789895@163.com (J.C.); taifang19820118@163.com (Q.L.); shaopeng@whpu.edu.cn (S.Z.); 12316@whpu.edu.cn (S.C.); 2 School of Life Science and Technology, Wuhan Polytechnic University, Wuhan 430023, China; 3 National R&D Center for Se-Rich Agricultural Products Processing Technology, Wuhan Polytechnic University, Wuhan 430023, China

**Keywords:** *Cyclocarya paliurus*, selenium polysaccharide, exogenous selenium fortification, antioxidant, immune regulation

## Abstract

In this study, we extracted, separated, and purified polysaccharides from Se-enriched *Cyclocarya paliurus* (Se-CPP-1) and compared them with their non-Se-enriched counterparts (CPP-1) to investigate the impact of selenium on their structural and functional properties. Structural characterization by HPLC, GC-MS, and SEM revealed that Se-CPP-1 is an acidic heteropolysaccharide with a lower molecular weight (76.6 vs. 109.22 kDa), smaller particle size (418.22 vs. 536.96 nm), and higher negative zeta potential (−43.15 vs. −21.29 mV), indicating enhanced colloidal stability. SEM imaging further demonstrated a distinctive flaky morphology in Se-CPP-1. Functional assays showed that Se-CPP-1 significantly outperformed CPP-1 in scavenging free radicals (DPPH/ABTS), stimulating RAW264.7 macrophage proliferation (CCK-8 assay), enhancing phagocytic activity, and promoting NO secretion. These improvements were attributed to selenium-induced modifications in polysaccharide conformation and surface properties. Our findings highlight the potential of selenium fortification in developing high-efficacy *C. paliurus* polysaccharides for antioxidant and immunomodulatory applications.

## 1. Introduction

As a traditional medicinal and edible plant, *Cyclocarya paliurus* (also known as sweet tea tree) has increasingly gained attention in the field of functional food ingredients in recent years. Its leaves, serving as natural tea materials, are not only rich in various nutrients beneficial to the human body but also exhibit unique pharmacological effects, making *C. paliurus* a functional food ingredient with high potential. This rare tree species is unique to China, predominantly found in various provinces south of the Yangtze River, including Anhui, Jiangxi, Hunan, Sichuan, and Guizhou. It is renowned for its abundant bioactive compounds, making it a medicinal and nutritious plant native to China [1]. The leaves comprise polysaccharides, flavonoids, triterpenoid saponins, and trace elements which play significant roles in health, including hypoglycemic, hypolipidemic, and antioxidant activities [2,3,4].

Selenium (Se) is a crucial trace element that offers numerous advantages for plants [5]. It not only facilitates the growth and development of plants but also augments their antioxidant defense mechanisms. Selenium also modulates chlorophyll synthesis and protein metabolism, enhancing the physiological activity of plants and the quality of the harvest [6,7,8]. There are two main forms of Se: inorganic and organic [9]. Se in plants primarily exists in organic forms, which involve selenoproteins and Se-polysaccharides [10,11,12]. The human body more readily absorbs and utilizes these organic selenium compounds. Conversely, inorganic selenium (e.g., sodium selenite, sodium selenate) may induce toxicity upon absorption and exhibit reduced bioavailability. Selenium in plants has enhanced bioavailability and reduced toxicity, making it more appropriate for human absorption and utilization. Consequently, the selenium needs of the human body can be fulfilled more effectively by the consumption of selenium derived from plants.

The biological activity of *C. paliurus* is greatly increased by selenium enrichment, which encourages its use in the production of medicinal foods and medications [13]. The characteristic polysaccharides of *C. paliurus* (CPP) contain a relatively high proportion of galacturonic acid and galactose and display modifiable bioactivity profiles. Chemical modifications including acetylation, phosphorylation, and selenization have been systematically shown to improve both bioactivity and bioavailability [14,15,16]. *C. paliurus* polysaccharides can alleviate intestinal inflammation by modulating the expression of inflammatory cytokines [17]. Specifically, phosphorylated CPP exhibit enhanced antitumor effects against colorectal cancer CT-26 cells compared to native CPP, with the phosphate group identified as a critical functional moiety [18]. Selenopolysaccharides synergistically combine selenium’s redox properties with polysaccharide bioactivity. Empirical evidence confirms their dual advantages: (1) apricot mushroom-derived selenopolysaccharides (Se-SPEP) demonstrate superior antioxidant capacity relative to their non-selenized counterparts, alongside heavy metal toxicity mitigation potential [19], while (2) selenium-enriched morel polysaccharides (Se-MPS) enhance macrophage immunological responses through TLR4-TRAF6-MAPKs-NF-κB pathway activation [20], providing mechanistic insights applicable to *C. paliurus* polysaccharide research.

Selenium, as an essential trace element for the human body, has derivatives that possess significant application value in the fields of functional foods, nutritional supplements, and disease prevention. In recent years, selenium-enriched agricultural products developed through exogenous selenium fortification technology, such as selenium-enriched yeast and selenium-enriched grains, have been successfully applied in selenium supplementation interventions for sub-healthy populations. Moreover, the selenium modification of plant polysaccharides has become a research hotspot in the development of functional foods due to its unique biological activities. It is worth noting that most of the existing studies focus on traditional selenium-enriched crops (such as *Astragalus membranaceus* and *Ganoderma lucidum*), while research on the selenium fortification of *C. paliurus*, a plant with both medicinal and edible properties, remains scarce. This study sought to examine the impact of selenium foliar application on *C. paliurus*, concentrating on the extraction, separation, purification, and identification of polysaccharides (CPP-1) and selenium polysaccharides (Se-CPP-1), while also performing the assessment of their biological activities. Right now, there are few publications regarding the active constituents of selenium-enriched *C. paliurus*. The experimental results demonstrate that selenium enrichment increases the biological activity of *C. paliurus*. The findings offer substantial support for the research and product development of active constituents in selenium-enriched *C. paliurus*. They provide a theoretical foundation for the advancement of products with improved nutritional value and health benefits, hence facilitating their prospective use in health food and pharmaceutical development.

## 2. Materials and Methods

### 2.1. Materials and Chemicals

This study utilized four-year-old *C. paliurus* seedlings sourced from Wufeng Mountain in Yichang City, Hubei Province. The experimental site was established in Sanhua Town, Xishui County, Huanggang City, Hubei Province, where the soil selenium content is less than 0.3 mg/kg. The seedlings were divided into two groups: the treatment group received a foliar spray of 160 mg/L sodium selenite, while the control group was treated with ddH2O. Fifteen days post-spraying, fresh leaves were collected, dried, and powdered, subsequently passed through an 80-mesh sieve for storage and future use. Trifluoroacetic acid, DMSO (chromatographic purity, purchased from Shanghai Amper experiment Technology Co., Ltd., Shanghai, China), monosaccharide standard (purchased from Sigma, Burlington, MA, USA), LPS, a DPPH kit, an ABTS kit, a CCK-8 kit (all purchased from Beijing Solarbio Science and Technology Co., Ltd., Beijing, China), DMEM medium, RAW cell culture medium, 1 × PBS buffer, neutral red, and a Nitric Oxide Detection Kit (Shanghai Beyotime, Shanghai, China) were used in the experiments. All the chemicals used were analytically pure.

### 2.2. Extraction and Purification Process

According to existing methods, CPP-1 and Se-CPP-1 are extracted from the leaves of *C. paliurus* using a water extraction method followed by alcohol precipitation [21]. After passing through an 80-mesh sieve, the dried powder of *C. paliurus* was extracted with deionized water at 90 °C for 2 h. Following centrifugation of the extract, the supernatant was subjected to AB-8 resin to remove pigments. The post-column extract was then treated with Sevage reagent to eliminate proteins and subsequently mixed with an equal volume of 80% anhydrous ethanol for alcohol precipitation to obtain crude polysaccharides. The crude polysaccharides, after being washed with anhydrous ethanol 4–5 times, underwent freeze-drying. Purification of the crude polysaccharides was conducted using the anionic exchanger DEAE-52 cellulose, followed by gradient elution with NaCl solutions of varying concentrations, with the eluates being collected. The eluates under the main elution peak were combined and concentrated. After dialysis and freeze-drying, purified CPP-1 and Se-CPP-1 were obtained. The selenium content was quantified using atomic fluorescence spectrometry. In this study, the determination of polysaccharide content was performed using the phenol-sulfuric acid method.

### 2.3. Carbohydrate Analysis

Molecular weight: The polysaccharide was solubilized in a 0.1 M NaNO_3_ aqueous solution containing 0.02% (*w*/*w*) NaN_3_ to attain a final concentration of 1 mg/mL. The solution was subsequently filtered using a filter with a pore size of 0.45 µm. A cutting-edge gel permeation chromatography (GPC) system featuring a refractive index (RI) detector and a multi-angle laser light scattering (MALLS) detector was utilized for analysis. The experimental parameters were as follows: column temperature of 45 °C, injection volume of 100 µL, mobile phase comprising 0.1 M NaNO_3_ with 0.02% NaN_3_, flow rate of 0.6 mL/min, and isocratic elution for 75 min.

Monosaccharide composition: Polysaccharide samples were acidolyzed in a 2 M solution of trifluoroacetic acid (TFA) at high temperatures. They were blown dry with nitrogen, washed multiple times with methanol to eliminate contaminants, and then redissolved in sterile water prior to testing. An electrochemical detector and Dionex CarboPac PA20 column were employed to evaluate monosaccharide fractions using a Thermo ICS 5000+ ion chromatography system. The injection volume was 5 μL, the mobile phase flow rate was 0.5 mL/min, and the column temperature was kept at 30 °C.

Spectral analysis: The materials were analyzed with a UV-visible spectrophotometer (UV-vis, Thermo, Waltham, MA, USA) within the spectrum of 190–600 nm [19].

The samples (CPP-1 and Se-CPP-1) were analyzed using an FT-IR spectrometer (FT-IR, Perkin Elmer, Waltham, MA, USA) within the region of 4000–400 cm^−1^ via the KBr compression method [22,23].

The particle size and zeta potential: The particle size and zeta potential of CPP-1 and Se-CPP-1 were measured using a particle size and zeta potential analyzer, with all samples at a concentration of 1 mg/mL.

Microstructure and properties: To examine the shape and crystal structure of polysaccharides before and following selenium enrichment, this study used SEM (Zeiss Merlin Compact, ZEISS, Oberkochen, Germany) and AFM (Dimension ICON, Bruker, Billerica, MA, USA). CPP-1 and Se-CPP-1 were passed through a 100-mesh filter, and a small portion was placed on conductive carbon tape, coated with gold, and subsequently examined and photographed using an electron microscope. A 10 μg/mL solution of CPP-1 and a 10 μg/mL solution of Se-CPP-1 were separately prepared and filtered through a 0.45 μm membrane.

The solution was then applied on a new mica surface and dried at room temperature for 12 h. AFM was utilized to assess the ultrastructure of CPP-1 and Se-CPP-1. The crystal structure was examined utilizing an X’ Pert Pro X-ray diffractometer (XRD, Shimadzu XRD-600, Tokyo, Japan) within a scanning range of 5° to 60°. The purified CPP-1 and Se-CPP-1 samples were desiccated and pulverized, sieved through a 100-mesh filter, weighed at 20 mg on a carrier platform, uniformly pressed and distributed, and subsequently analyzed using the apparatus.

### 2.4. Antioxidant Capacity Assessment

The DPPH and ABTS radical scavenging assays are extensively utilized for then quantitative determination of the in vitro antioxidant capacity of biological samples, pure compounds, and extracts. The radical scavenging abilities of CPP-1 and Se- CPP-1 towards DPPH and ABTS radicals were evaluated using previously established methodologies. Test objects (CPP-1 and Se-CPP-1) and vitamin C (Vc) were weighed separately and dissolved in ultrapure water. After thorough mixing, the sample solutions were diluted to six different concentrations for subsequent use. For the DPPH assay, various reagents were added according to the manufacturer’s instructions (Beijing Solarbio Science & Technology Co., Ltd., Beijing, China), vortexed for mixing, and incubated in the dark at room temperature for 30 min. Each group was set up in triplicate, and the absorbance was measured at 515 nm, recorded as A0, A, and A1, respectively. Similarly, for the ABTS assay, different reagents were added following the manufacturer’s protocol, thoroughly mixed, and incubated in the dark at room temperature for 6 min. The absorbance was then measured at 405 nm. The scavenging rate was calculated using the following formula.

(1) $$\mathrm{DPPH}/\mathrm{ABTS}\;\mathrm{Radical}\;\mathrm{Scavenging}\;\mathrm{Rate}\;(D\%)=[\frac{A0-(A-A1)}{A0}]\times 100\%,$$

In this case, the absorbance of the sample group is *A*, the absorbance of the control group is *A*1, and the absorbance of the blank group is *A*0.

### 2.5. Immune Activity Assessment

#### 2.5.1. Cell Cultures

Mouse macrophage RAW 264.7 cells were cultured in DMEM medium supplemented with 10% FBS at 37 °C in a cell culture incubator with 5% CO_2_.

#### 2.5.2. Cell Viability Assay

The proliferative capacity of CPP-1 and Se-CPP-1 was assessed using the CCK-8 test. RAW 264.7 cells were inoculated at a density of 10^4^ cells/mL in a 96-well plate and incubated for 24 h. Different concentrations of CPP-1 and Se-CPP-1 (25, 50, 100, 200, 400, and 800 µg/mL) were added to each well and thereafter incubated for 24 h. In addition, CCK-8 reagent was added to each well and incubated for two hours. Absorbance was measured at 450 nm using a microplate reader (PerkinElmer, Richmond, VA, USA).

#### 2.5.3. Cell Phagocytosis Assay

Cell phagocytic capacity was assessed using a neutral red uptake test. CPP-1 and Se-CPP-1 at varying concentrations (25, 50, 100, 200, 400, and 800 µg/mL) were inoculated into a 96-well plate and incubated for 24 h. A neutral red solution was thereafter introduced to each well and incubated for one hour. Then, the supernatant was discarded, and the wells were rinsed three times with PBS to eliminate the neutral red solution. Each well received a prepared cell lysis solution, which was then incubated at 37 °C for two hours. The absorbance was quantified at 490 nm utilizing a microplate reader (PerkinElmer, VA, USA).

#### 2.5.4. Analysis of NO

RAW 264.7 macrophage cells’ nitric oxide (NO) concentration was quantified using the Griess reagent. Different amounts of CPP-1 and Se-CPP-1 (25, 50, 100, 200, 400, and 800 µg/mL) were co-cultured with RAW 264.7 macrophage cells on a 96-well plate for 48 h. Test concentrations were established as specified, and blank and negative control groups were created. The well’s supernatant was moved to a fresh 96-well plate, combined with a predetermined amount of Griess reagent, and then allowed to sit at room temperature for ten minutes. The absorbance was subsequently quantified at 540 nm utilizing a microplate reader (PerkinElmer, VA, USA). A standard curve was established using the kit’s guidelines (Shanghai Beyotime) to quantify the NO content.

### 2.6. Statistical Analysis

The data were processed using SPSS 20.0 software, and within-group comparisons were conducted by one-way ANOVA, with results displayed using GraphPad Prism 10.1. Data are expressed as mean ± standard deviation. Significant results are defined as *p* < 0.05.

## 3. Results

### 3.1. Extraction, Separation, and Purification of CPP-1 and Se-CPP-1

**Figure 1:**
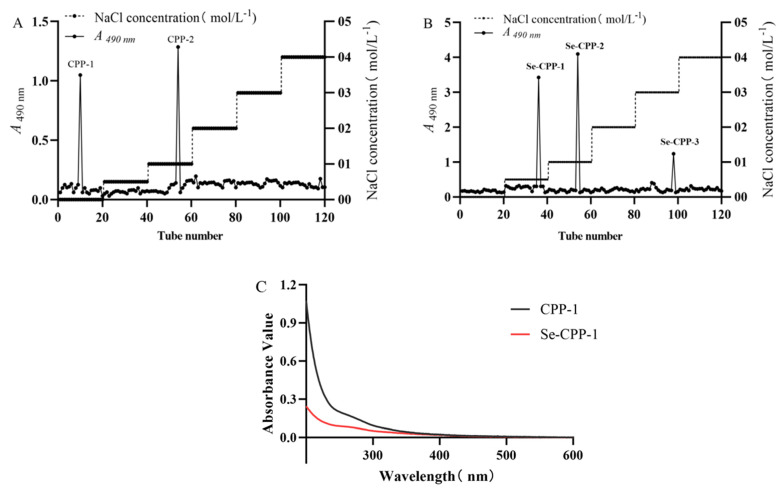
Purification of polysaccharides and selenium polysaccharides. (**A**,**B**) Elution profiles of Se-CPP-1 and CPP-1; (**C**) UV results of Se-CPP-1 and CPP-1.

Crude polysaccharides were extracted and isolated from two types of *C. paliurus* powder sample, one selenium-enriched and the other non-selenium-enriched. To achieve homogeneity, the polysaccharides obtained needed to undergo further purification subsequent to their preliminary separation. Further purification was achieved using gradient elution with varying concentrations of NaCl on a DEAE-52 column, yielding CPP and Se-CPP. For CPP, two major components were recovered at NaCl concentrations of 0.1 mol/L and 0 mol/L, designated as CPP-1 and CPP-2, respectively (Figure 1A). Similarly, Se-CPP yielded two primary elution fractions at NaCl concentrations of 0.05 mol/L and 0.1 mol/L, named Se-CPP-1 and Se-CPP-2, respectively (Figure 1B). Subsequent research focused on CPP-1 and Se-CPP-1, as they exhibited the highest total polysaccharide yields among the eluted fractions from the two polysaccharide groups. The lack of notable absorption peaks in the UV spectral region of 250–300 nm indicates that CPP-1 and Se-CPP-1 are devoid of proteins and nucleic acids, allowing for further structural and morphological investigations of CPP-1 and Se-CPP-1.

### 3.2. Effect of Selenisation on Physicochemical Properties

#### 3.2.1. Molecular Weight and Monosaccharide Composition Analysis

**Figure 2:**
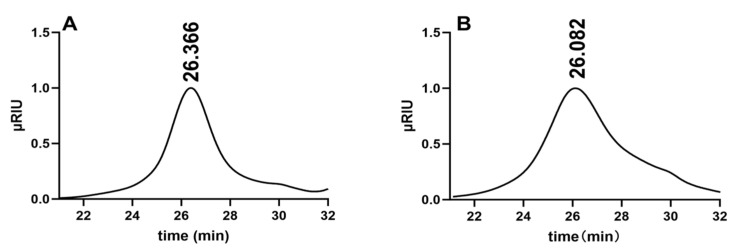
Molecular weight effects. CPP-1 (**A**); Se-CPP-1 (**B**).

Compositional analysis constitutes a pivotal phase in the quality control standards for polysaccharides, providing indispensable information regarding their properties [24]. Figure 2 illustrates the results of molecular weight determination for CPP-1 and Se-CPP-1. A single dominant absorption peak was detected for both, indicating the high purity and excellent homogeneity of CPP-1 and Se-CPP-1. Through software calculations, the molecular weights of CPP-1 and Se-CPP-1 were determined to be 109.22 kDa and 76.662 kDa, respectively. Notably, the selenium-modified *C. paliurus* polysaccharide (Se-CPP-1) exhibits a lower molecular weight, suggesting enhanced bioavailability and ease of absorption by the organism. Selenium modification leads to a significant reduction in the molecular weight of lentinan and an increase in its chain rigidity, resulting in a more extended conformation in solution. This in turn causes a decrease in the cellular uptake rate and a change in the uptake pathway [25]. The monosaccharide composition results are tabulated in Table 1. CPP-1 is composed of galactose, arabinose, rhamnose, mannose, glucuronic acid, and galacturonic acid. In contrast, Se-CPP-1 comprises galactose, arabinose, glucose, fucose, galacturonic acid, and glucuronic acid. Notably, both share the constituents of galactose, arabinose, glucose, galacturonic acid, and glucuronic acid, which aligns with prior research findings. The relative content of galacturonic acid in CPP-1 and Se-CPP-1 was measured at 6.10% and 19.81%, respectively, thereby classifying them as acidic polysaccharides [26]. Rhamnose and galacturonic acid influence the antioxidant effectiveness of polysaccharides [27]. Li et al. [28] verified that the contents of arabinose and galacturonic acid affected the scavenging ability of polysaccharides against DPPH radicals, and the contents of galacturonic acid and uronic acid influenced their scavenging ability against hydroxyl radicals. Se-CPP-1 has more glucuronic acid than CPP-1, making it more antioxidant.

#### 3.2.2. Determination of Particle Size, Zeta Potential, and Selenium Content

To further investigate the structures of CPP-1 and Se-CPP-1, this study determined their particle sizes and selenium contents. The total selenium content of CPP-1 and Se-CPP-1 was 1.26 mg/kg and 1.73 mg/kg, respectively, and the selenium content of Se-CPP-1 was increased by 0.47 mg/kg compared with that of CPP-1.

Based on dynamic light scattering (DLS) results, the particle sizes of CPP-1 and Se-CPP-1 were found to be 536.96 nm and 418.22 nm, respectively (Table 2). The results indicated no significant changes in either sample over a 30-day period, suggesting high stability for both CPP-1 and Se-CPP-1. In dispersion systems, the ζ potential serves as a crucial indicator for assessing particle stability and electrostatic interactions [29]. The zeta potential values are listed in Table 2. Specifically, CPP-1 and Se-CPP-1 exhibited zeta potentials of −21.29 mV and −43.15 mV, respectively, indicating that Se-CPP-1 bears a high negative charge and possesses excellent stability in solution.

### 3.3. Structural Effects of Selenisation

**Figure 3:**
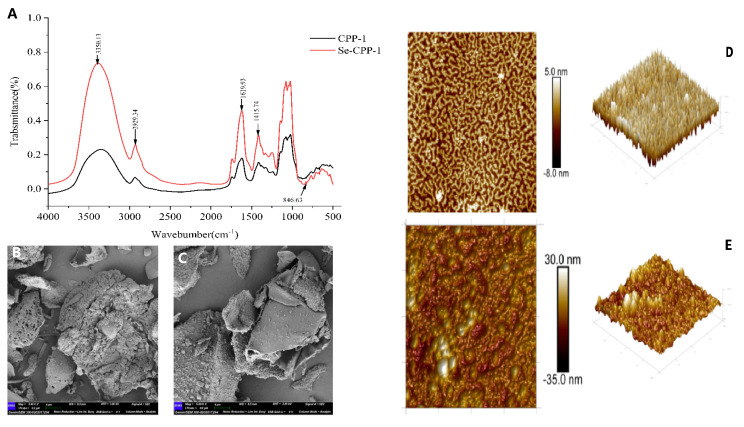
Structural changes of CPP-1 and Se-CPP-1. FT-IR results (**A**); scanning electron microscopy image of CPP-1 (**B**); scanning electron microscopy image of Se-CPP-1 (**C**); AFM results of CPP-1 (**D**); AFM results of Se-CPP-1 (**E**).

The biological activity of polysaccharides is based on their structure. Chemical composition, molecular weight, and monosaccharide composition are among the distinctive architectures of polysaccharides that are typically linked to their biological function [26,30]. The biological activity of both CPP-1 and Se-CPP-1 is increased when they combine to produce organic molecules [31]. Higher-relative-molecular-weight polysaccharides are less soluble, which impacts their bioavailability, according to some studies; conversely, low-molecular-weight polysaccharides can also affect their biological activity [32]. Their structural properties greatly influence their biological activity, and Fourier transform infrared spectroscopy (FT-IR) is a useful technique for describing the functional groups of polysaccharides [28]. Both Se-CPP-1 and CPP-1 are high-molecular-weight polysaccharides, and additional study of their functional aspects is made easier by their structural features. The CPP-1 and Se-CPP-1 spectra in the 4000–400 cm^−1^ region are displayed in Figure 3A. The stretching vibration of -OH is responsible for a high absorption peak at 3350.13 cm^−1^, whereas the C-H stretching vibrations of the methyl and methylene groups are responsible for the peaks at 2929.34 cm^−1^. The stretching vibration peak of the carboxyl C = O is situated at 1619.93 cm^−1^.

Furthermore, 1415.74 cm^−1^ is the distinctive absorption peak of C-H bending. In conclusion, the infrared spectral properties of polysaccharides are displayed by the selenium-modified Se-CPP-1, and the O-Se-O link is responsible for the new absorption peak at 846.63 cm^−1^. These outcomes support the polysaccharide’s successful selenium alteration, which is in line with Gu et al.’s [33] findings. SEM, or scanning electron microscopy, is a powerful tool for the high-resolution observation and analysis of molecular surface morphology. Exogenous selenium significantly affects the shape of polysaccharides, as seen in Figure 3. At a magnification of 5.0 K, Se-CPP-1 has a smooth surface and an irregular lamellar morphology, whereas CPP-1 has an uneven and rough surface with an irregular spherical cross-linked structure (Figure 3B,C). Conversely, it is discovered that Se-CPP-1 is a loose, porous, granular cluster aggregate with a much smaller particle size. Huang [34] came to the same findings. The SEM results provide additional confirmation of the particle size determination based on the aforementioned events. Consequently, it is postulated that the selenium in Se-CPP-1 might function as a hydrophobic binding site, causing the extended sugar chains to fold, resulting in morphological alterations and a decrease in particle size.

Atomic force microscopy (AFM) was used to examine the surface structure of polysaccharides in more detail. The advantage of AFM over SEM is its ability to observe the surface morphology of substances in physiological settings [35]. CPP-1 exhibits molecular aggregation as an irregular fibrous network made up of randomly ordered linear chains, as seen in Figure 3D,E. Meanwhile, there are several spherical clusters in the structure of Se-CPP-1, which indicate greater molecular aggregation. This indicates that compared to CPP-1, Se-CPP-1 exhibits more molecular aggregation. Subsequent analysis reveals that the polysaccharide chains of Se-CPP-1 have a longitudinal height of roughly 30.0 nm, whereas those of CPP-1 are about 5.0 nm in height. According to the infrared results, polysaccharide chains typically range in size from 0.1 to 1.0 nm. This phenomenon could be caused by polysaccharide molecules entangling with one another, as evidenced by the broad peak in the 3300–3400 cm^−1^ range for Se-CPP-1 and the 0.1–1.0 cm^−1^ range for CPP-1.

**Figure 4:**
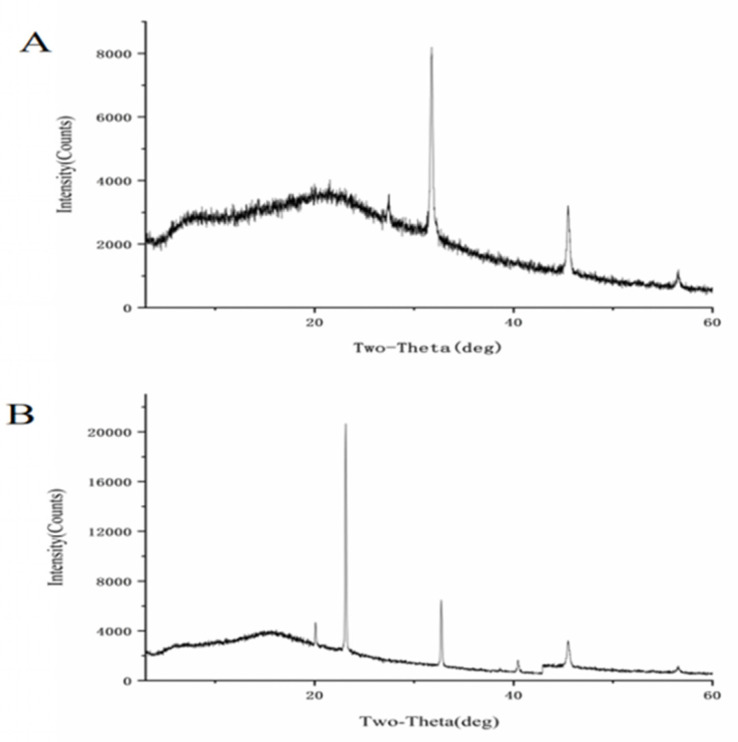
XRD patterns of CPP-1 (**A**) and Se-CPP-1 (**B**).

Using X-ray diffraction (XRD) patterns, the polysaccharide crystalline structure and morphology can be identified [36]. The findings are displayed in Figure 4, where the diffraction intensity diagrams of CPP-1 and Se-CPP-1 both show large diffraction peaks. The higher XRD peak intensity of selenized polysaccharides compared to native polysaccharides may be attributed to the introduction of selenium. Selenium incorporation could potentially alter the glycosidic bond linkages or intermolecular interactions of the polysaccharides. The XRD spectrum of CPP-1 is displayed in Figure 4A, where a more noticeable crystalline peak is located at the diffraction angle (2θ) of 32 °C. The XRD spectrum of Se-CPP-1 is displayed in Figure 4B, where a prominent crystalline peak is located at the diffraction angle (2θ) of 23 °C. In conclusion, crystalline peaks of Se are visible in both CPP-1 and Se-CPP-1. Compared with CPP-1, Se-CPP-1 has fewer diffraction peaks. Furthermore, the investigation shows that the diffraction peaks of selenium-modified polysaccharides are greater than those of the original polysaccharides.

### 3.4. Effect of Selenisation on Antioxidant Activity

**Figure 5:**
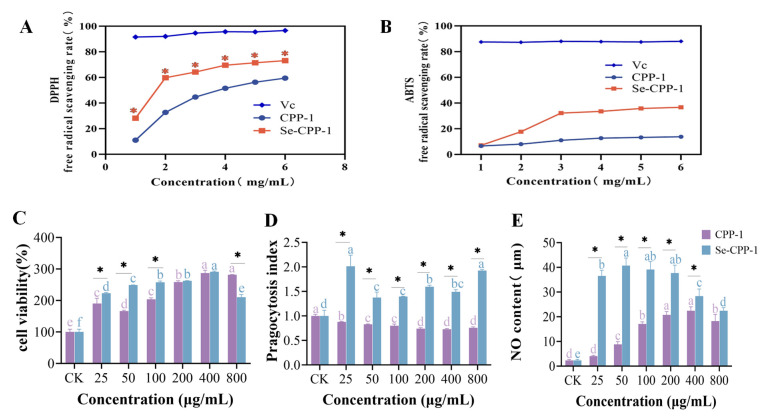
In vitro comparison of the activity between polysaccharides and selenium polysaccharides. DPPH radical scavenging experiment (**A**) and ABTS radical scavenging experiment (**B**); cytotoxicity experiment on RAW 264.7 cells (**C**); phagocytic activity of macrophages (**D**); nitric oxide secretion (**E**). Different letters represent changes between different concentrations of the same sample. * indicates variations between samples with the same concentration. Statistical significance was assessed using Duncan’s multiple-value test (*p* < 0.05).

The DPPH radical is a nitrogen-centered free radical that is extremely persistent and one of the most significant markers of antioxidant capability in plants, animals, and people. It is frequently employed in research on medications, nutraceuticals, and foods high in antioxidants [37]. Both polysaccharides demonstrated strong DPPH radical scavenging ability in a dose-dependent manner, as shown in Figure 5A. CPP-1 (IC50 = 3.953 mg/mL) and Se-CPP-1 (IC50 = 1.861 mg/mL) demonstrated scavenging rates of 56.02% and 72.06%, respectively, at a concentration of 6 mg/mL. Across all concentration ranges, Se-CPP-1 consistently outperformed CPP-1 in terms of scavenging.

The foundation of the ABTS radical scavenging assay is the idea that antioxidants present in the samples can efficiently interact with ABTS radicals, causing the reaction system to become noticeably discolored. A quantitative way to measure antioxidant capacity is provided by this interaction, which causes a noticeable drop in absorbance at 405 nm that is positively connected to the extent of radical scavenging. Figure 5B exhibits the ABTS scavenging assay results. At concentrations between 1 and 2 mg/mL, there was no discernible difference in the ABTS radical scavenging rate between CPP-1 and Se-CPP-1. This could be because there were not enough reducing groups available to cause ABTS dissociation at low doses. The scavenging rates, however, varied with increasing CPP-1 and Se-CPP-1 concentrations, reaching 15.78% and 38.96% at 6 mg/mL, respectively. Therefore, Se-CPP-1 outperformed CPP-1 in terms of ABTS radical scavenging activity. These results align with those of other selenium-based polysaccharide studies [38].

The biological activity of polysaccharides is affected by their physicochemical qualities and structure, such as monosaccharide composition, molecular weight, glyoxylate content, and protein content. Polysaccharides with a higher glucuronic acid concentration have greater antioxidant capability, but selenopolysaccharides have a higher antioxidant capacity [39]. Thus, selenium may alter *C. paliurus* polysaccharides and lower their molecular weights, increasing their antioxidant activity. Furthermore, selenium in selenopolysaccharides activates the hydrogen atoms of the allosteric carbons, enhancing the polysaccharides’ ability to supply energy to hydrogen atoms and increasing their free radical scavenging capacity [19]. Research has revealed that among *Polygonatum sibiricum* polysaccharides with similar monosaccharide compositions, polysaccharides with lower molecular weights exhibit better antioxidant effects. This might be attributed to the fact that polysaccharides with higher molecular weights have a larger volume. This makes it difficult for them to cross the cell membrane and be utilized by the organism [40].

### 3.5. Effect of Selenisation on Immunological Activity

#### 3.5.1. Cell Proliferation

In the CCK-8 experiment, the more cells there are and the darker the color during incubation, the higher the cell multiplication rate; on the other hand, the color lightens if the cytotoxicity increases. Cell survival, phagocytosis, and NO secretion of RAW 264.7 cells were used to assess the impact of CPP-1 and Se-CPP-1 on the immunomodulatory activities of these cells. The CCK-8 assay was employed to examine the detrimental effects of different dosages of CPP-1 and Se-CPP-1 on RAW 264.7 macrophages (Figure 5C). The incorporation of different amounts of CPP-1 and Se-CPP-1 significantly enhanced the survival rate of RAW 264.7 cells, as depicted in the image. Se-CPP-1 significantly enhanced the proliferation of RAW 264.7 cells relative to CPP-1; however, cell viability decreased at a concentration of 800 μg/mL. In the 25–800 μg/mL range, the cell survival rates of Se-CPP-1 were 23.1%, 248.8%, 257.9%, 262.2%, 290.9%, and 210.1%, respectively. The experimental findings demonstrated that Se-CPP-1 significantly enhances cell proliferation in a dose-dependent fashion. Considering all of this, the next tests used a concentration range of 25 to 800 μg/mL.

#### 3.5.2. Phagocytic Activity Assay

The phagocytic capacity demonstrated by macrophages occupies a pivotal role in the host defense mechanisms, serving as a crucial biological marker for assessing the state of cellular activation. Activated macrophages possess the ability to engulf pathogens and interact with other immune cells, thereby enabling fine-tuned regulation of immune responses [41]. Figure 5D illustrates that cells treated with Se-CPP-1 exhibited enhanced phagocytic activity and significantly stimulated phagocytosis of RAW 264.7 cells across various concentrations (*p* < 0.05) in comparison to the blank and CPP-1 groups, indicating that selenium-enriched polysaccharides augment phagocytosis in RAW 264.7 macrophages. The phagocytic activity of Se-CPP-1 reached its peak at a concentration of 25 μg/mL. Previous studies found that selenium-modified polysaccharides dramatically boosted the activity of RAW 264.7 macrophages. Enhanced phagocytosis by phagocytes has been demonstrated as a mechanism by which selenium-modified polysaccharides bolster immunity.

#### 3.5.3. Measurement of NO Content

Nitric oxide (NO) may regulate many physiological and pathological responses in vivo, stimulate lymphocyte proliferation, and play a crucial role in host defense [42]. Figure 5E shows that when murine macrophages were stimulated with varying doses of both polysaccharides, NO secretion increased considerably. The selenylate polysaccharide greatly enhanced the secretion of NO by RAW 264.7 macrophages. The maximal concentration of NO rose from 2.29 ± 0.39 μM to 40.75 ± 0.96 μM (50 µg/mL) and 22.41 ± 1.4 μM (400 µg/mL) when subjected to varying dosages of Se-CPP-1 and CPP-1 in comparison to the control group. This indicates that Se-CPP-1 may have an immunological function by promoting NO secretion from macrophages. Numerous intracellular signaling pathways, including MAPK and NF-κB, are affected by polysaccharides. Polysaccharides can independently or jointly stimulate the activation of RAW264.7 cells and the release of cellular immune mediators by inducing the release of cytokines (IL-6, IL-10) and tumor necrosis factor (TNF), which subsequently activates other immune cells. According to Qian et al. [20], selenium-enriched morel polysaccharides (Se-MPS) triggered the TLR4-TRAF6-MAPKs-NF-κB signaling pathway, which in turn stimulated the immunological responses of RAW 264.7 macrophages and greatly increased phagocytosis.

Selenium-modified *Ganoderma lucidum* microsellar polysaccharides markedly enhanced the generation of nitric oxide (NO), in addition to cytosolic activity and phagocytosis in RAW 264.7 cells [43]. Qiu [44] and colleagues quantitatively altered garlic polysaccharides with selenium and discovered that selenium supplementation markedly improved the immunological efficacy of garlic polysaccharides obtained from chicken lymphocyte samples. Chen et al. [45] discovered that the monosaccharide composition of polysaccharides correlates with their immunomodulatory effect, and that the presence and concentration of monosaccharides such as galactose, glucose, arabinose, and mannose may affect immunological activity.

## 4. Conclusions

In this study, a selenium-enriched *C. paliurus* polysaccharide (Se-CPP-1) was successfully developed via exogenous selenium fortification. Novel structural advantages of Se-CPP-1 were revealed, including a reduced molecular weight (76.6 kDa vs. 109.22 kDa), a smaller particle size (418.22 nm vs. 536.96 nm), and enhanced stability attributed to the increased negative charge (−43.15 mV vs.−21.29 mV). Compared with the native *C. paliurus* polysaccharide-1 (CPP-1), this selenium-modified polysaccharide exhibited superior biofunctional properties. Specifically, it demonstrated enhanced antioxidant activity, as evidenced by its scavenging ability against DPPH/ABTS radicals. Moreover, it promoted the proliferation, phagocytosis, and nitric oxide (NO) release of RAW264.7 macrophages, indicating significant immunomodulatory effects. Although these findings pioneered the establishment of the structure–activity relationship of selenium-engineered plant polysaccharides and provided a technological paradigm for the development of functional nutraceuticals, there are still limitations in clarifying the exact chemical speciation of selenium and validating its efficacy in vivo. Future research should employ advanced mass spectrometry to decipher the binding patterns of selenium and conduct pre-clinical trials to optimize the therapeutic dosage. This research not only offers a new direction for the further development of *C. paliurus* polysaccharides but also provides a reference for the selenium enrichment studies of other plant polysaccharides.

## Figures and Tables

**Table 1:** The monosaccharide composition.

Fraction	Percentage Composition of Monosaccharides (%)								
	Fuc	Rha	Ara	Gal	Glc	Xyl	Man	Gal-UA	Glc-UA
CPP-1	0.00	4.23	30.09	44.90	8.65	1.69	2.00	6.10	2.34
Se-CPP-1	0.75	11.61	25.06	32.15	6.37	2.48	0.00	19.81	1.77

Gal: galactose; Ara: arabinose; Rha: rhamnose; Glc: glucose; Xyl: xylose; Fuc: fucose; Gal-UA: galacturonic acid; Glc-UA: glucuronic acid.

**Table 2:** The particle size, selenium content, and zeta potential of CPP-1 and Se-CPP-1.

	Particle Size d/nm	Zetapotential (mV)
CPP-1	536.96 ± 34.80 a	−21.29 ± 2.00 a
Se-CPP-1	418.22 ± 50.68 b	−43.15 ± 3.14 b

Data in the same line with different letters are significantly different (*p* < 0.05).

## Data Availability

The data presented in this study are available on request from the corresponding author.
